# The relationship between manual coordination and mental health

**DOI:** 10.1007/s00787-015-0732-2

**Published:** 2015-07-03

**Authors:** Liam J. B. Hill, Faisal Mushtaq, Lucy O’Neill, Ian Flatters, Justin H. G. Williams, Mark Mon-Williams

**Affiliations:** School of Psychology, University of Leeds, Leeds, LS2 9JT UK; Institute of Medical Sciences, University of Aberdeen, Clinical Research Centre, Royal Cornhill Hospital, Aberdeen, AB25 2ZH UK

**Keywords:** Psychomotor disorders, Developmental disorders, Motor skills, Child behaviour, Cross-sectional studies, Community psychiatry

## Abstract

Motor coordination impairments frequently co-occur with other developmental disorders and mental health problems in clinically referred populations. But does this reflect a broader dimensional relationship within the general population? A clearer understanding of this relationship might inform improvements in mental health service provision. However, ascertainment and referral bias means that there is limited value in conducting further research with clinically referred samples. We, therefore, conducted a cross-sectional population-based study investigating children’s manual coordination using an objective computerised test. These measures were related to teacher-completed responses on a behavioural screening questionnaire [the Strength and Difficulties Questionnaire (SDQ)]. We sampled 298 children (4–11 years old; 136 males) recruited from the general population. Hierarchical (logistic and linear) regression modelling indicated significant categorical and continuous relationships between manual coordination and overall SDQ score (a dimensional measure of psychopathology). Even after controlling for gender and age, manual coordination explained 15 % of the variance in total SDQ score. This dropped to 9 % after exclusion of participants whose SDQ responses indicated potential mental health problems. These results: (1) indicate that there is a clear relationship between children’s motor and mental health development in community-based samples; (2) demonstrate the relationship’s dimensional nature; and (3) have implications for service provision.

## Introduction

A clinically diagnosable mental health problem is present in approximately 10 % of children in the UK and 13–20 % in the USA [1, 2]. For a significant proportion of these individuals their mental health problems involve ‘Disorders of [their] Psychological Development’ [3]. The estimated prevalence of this specific disorder type in the USA is 7–8 % in 3–17 year olds [4]. Collectively these disorders are characterised by: (1) onset in infancy/childhood and (2) a negative effect on developmental functions related to maturation of the central nervous systems. They are alternatively labelled within ICD-10 as Pervasive and Specific Developmental Disorders and encompass pervasive developmental disorders (including autism) as well as specific disorders of speech and language, scholastic skills and motor function.

At first glance, the logic behind recognising motor function within this family of disorders may seem unclear. Indeed, until relatively recently (late 1980s) medicalization of coordination problems was highly contentious, with such problems more often attributed to inadequate teaching practices rather than to any underlying specific learning disability [5]. More recently, it has been suggested that children with coordination difficulties often present to occupational therapists, physiotherapists or paediatricians rather than child psychiatrists [6]. Thus, at a practical level problems with motor coordination appear distinct from those relating to mental health because treatment is accessed through markedly different routes.

Nonetheless, motor-coordination difficulties are noted to co-occur frequently alongside other neurodevelopmental disorders in clinically referred populations [7–9]. In fact, it has been suggested that co-occurring developmental disorders are more common than not in individuals diagnosed with developmental coordination disorder (DCD) [10, 11]. Broader mental health problems (such as anxiety and depression) are also noted to be common secondary consequences of living with a diagnosis of DCD [12, 13]. Meanwhile, relatively large studies of children (*n* > 200) diagnosed with pervasive developmental disorders, hyperkinetic disorders and/or speech, language or learning disabilities have indicated that between 20 and 40 % of these individuals have co-occurring coordination difficulties [14–16]. Smaller case–control studies (*n* < 59 cases) have repeatedly found that children diagnosed with autism spectrum disorder (ASD) [17, 18], attention deficit hyperactivity disorder (ADHD) [19, 20] and specific language impairment (SLI) [21, 22] exhibit evidence of impaired motor control abilities when compared to typically developing matched controls.

The frequency of such co-occurrences has led some to propose that common aetiologies may underpin motor coordination and other developmental disorders [15, 23, 24]. Similarly, it is noted that many of the biological risk factors associated with mental health problems, whether genetic, environmental or early developmental insult (e.g., prematurity) are also indicated as risk factors for developmental coordination difficulties [25, 26]. In summary, there is reasonable evidence to suggest that coordination difficulties and other mental health problems are often associated strongly with one another in clinically referred samples of children.

However, an appreciation of this capacity for co-occurrence appears to be largely absent from current clinical practice [10]. Surveys suggest a poor awareness amongst psychiatrists of the potential for coordination difficulties to exist in conjunction with other mental health problems [27], with few routinely assessing for coordination difficulties when making diagnoses. Developmental coordination disorder is routinely described in the literature as a ‘neglected’ or ‘hidden’ disability that is under-appreciated by education and health professions, particularly when other co-occurring developmental difficulties are also present [9–11, 28].

This lack of recognition may in part reflect a lack of confidence in the pre-existing research’s ability to define clearly the nature of the relationship between motor coordination and mental health. At present, the majority of evidence supporting an association between motor coordination and other mental health problems comes from studies that have utilised clinically referred samples (see preceding references). Whilst such studies typically demonstrate high degrees of co-occurring difficulties they have limited generalizability, due to problems with ascertainment and referral biases that are associated with such samples. Specifically, children referred for medical evaluation are likely to be those with the most severe and complex difficulties [29, 30]. Thus, the probability of these children meeting the criteria for multiple diagnoses is elevated—potentially giving an exaggerated impression of the relationships between different types of developmental impairment in the general population.

### Investigating relationships at a population level

Fewer studies have investigated co-occurrence using non-clinically acquired samples, free from referral and ascertainment bias. Two studies have looked at data from the Avon Longitudinal Study of Parents and Children (ALSPAC, *n* > 6000) and report an increased risk of parent-reported mental health difficulties, self-reported depression and poorer objectively assessed social-cognition skills in 7- to 9-year-old children categorised as having probable DCD [31, 32]. Earlier studies have also shown longitudinal relationships between coordination difficulties and anxiety and depression in non-clinically referred samples, particularly for males [33] and in the presence of co-occurring ADHD [34, 35]. Twin-studies have also provided insights into the aetiology underpinning certain types of co-occurrence. Some have found evidence for shared-genetic factors contributing to the co-occurrence of motor problems alongside both attention and anxiety problems [36, 37], whilst another reports non-shared environmental factors influencing the relationship between motor problems and co-occurring anxious and depressive symptomology [38]. This contradiction is possibly the result of differences in the criteria these studies used to define motor impairment. It could be argued though that these population-based studies all still share two important limitations: they define motor-skills proficiency dichotomously (as either impaired or otherwise) and use subjective assessments to make this classification.

### Investigating dimensional relationships

It is increasingly recognised that many of the symptoms that characterise mental health problems are in fact dimensionally rather than dichotomously distributed within the general population [39]. It has been proposed that identifying the degree to which motor difficulties associate dimensionally with other mental health problems could have important implications in relation to assessing and intervening to support children with mental health problems [9]. For example, in children with ADHD the level of motor impairment increases as a function of how many co-occurring disorders are present [40]. In a community-acquired sample, the risk of anxiety and depression was observed to be elevated in children with DCD, with this risk heightened further if they also had co-occurring ADHD [41]. Meanwhile, a longitudinal cohort study indicated that the extent to which children were delayed in their motor development during infancy (i.e., reaching fundamental ‘motor milestones’) predicted level of neuroticism in adulthood [42]—a risk factor for psychopathology.

At the same time, the question of what underlying causes may contribute to dimensionality within co-occurring developmental disorders has been scrutinised [43]. In a nationwide Swedish study of 6595 nine- and twelve-year-old twin pairs, Pettersson et al. identified a general genetic factor underpinning children’s liability to suffer from a wide range of non-specific neurodevelopmental symptoms using exploratory factor analysis. This factor was hierarchically related to three second-order genetic factors that explained proportionally less of the variance and appeared to cluster around sets of symptoms relating to specific syndromes (i.e., ‘inattention’; ‘learning problems’; and ‘tics and autism’). However, the attention paid to assessing motor impairments within this study was extremely limited, with evaluation of this aspect of development reliant upon a single item on the 53-item questionnaire used (“problems coordinating movements smoothly?” with three response levels: “Yes”, “No” or “Yes, to some extent”). Consequently, a more detailed assessment of whether a truly dimensional relationship specifically exists between motor coordination and other child mental health problems is required.

### Investigating motor function using objective assessments

A consistent weakness of existing community-based studies is their dependence on subjective and imprecise methods of assessing motor skills. Typically used are parent-report questionnaires [44] or standardised 1-to-1 assessments of motor competence [45]. Questionnaires have often been used out of necessity to make community-based sampling of large numbers of children practical. Meanwhile standardised 1-to-1 methods are often too time consuming for this purpose. They are also subject to inter-rater reliability issues [46]. Moreover, both approaches have been repeatedly criticised as being poorly suited to drawing meaningful distinctions between anything other than clearly typical versus atypical development [47, 48]. Technological advances are making it increasingly feasible to use brief, objective, computerised assessments to measure children’s motor skill, particularly manual coordination, in community-based settings [49]. Such methods are particularly well suited to conducting dimensional assessments of motoric ability, due to the increased fidelity and precision with which they can record individual participants’ responses.

To summarise, there exists a pressing need for more community-based investigation of the relationship between children’s motor coordination and other aspects of their mental health. In particular, research into the potentially dimensional nature of this relationship that utilises technological innovation to capture (with precision and objectivity) assessments of participant’s motor skill may provide valuable clinical insights. Consequently we conducted a cross-sectional study that utilised an objective computerised system to assess manual control within a sample of 4- to 11-year-old school children (all in mainstream education) and investigated the degree of association between this and a standardised dimensional assessment of children’s mental health.

## Method

### Participants

**Fig. 1:**
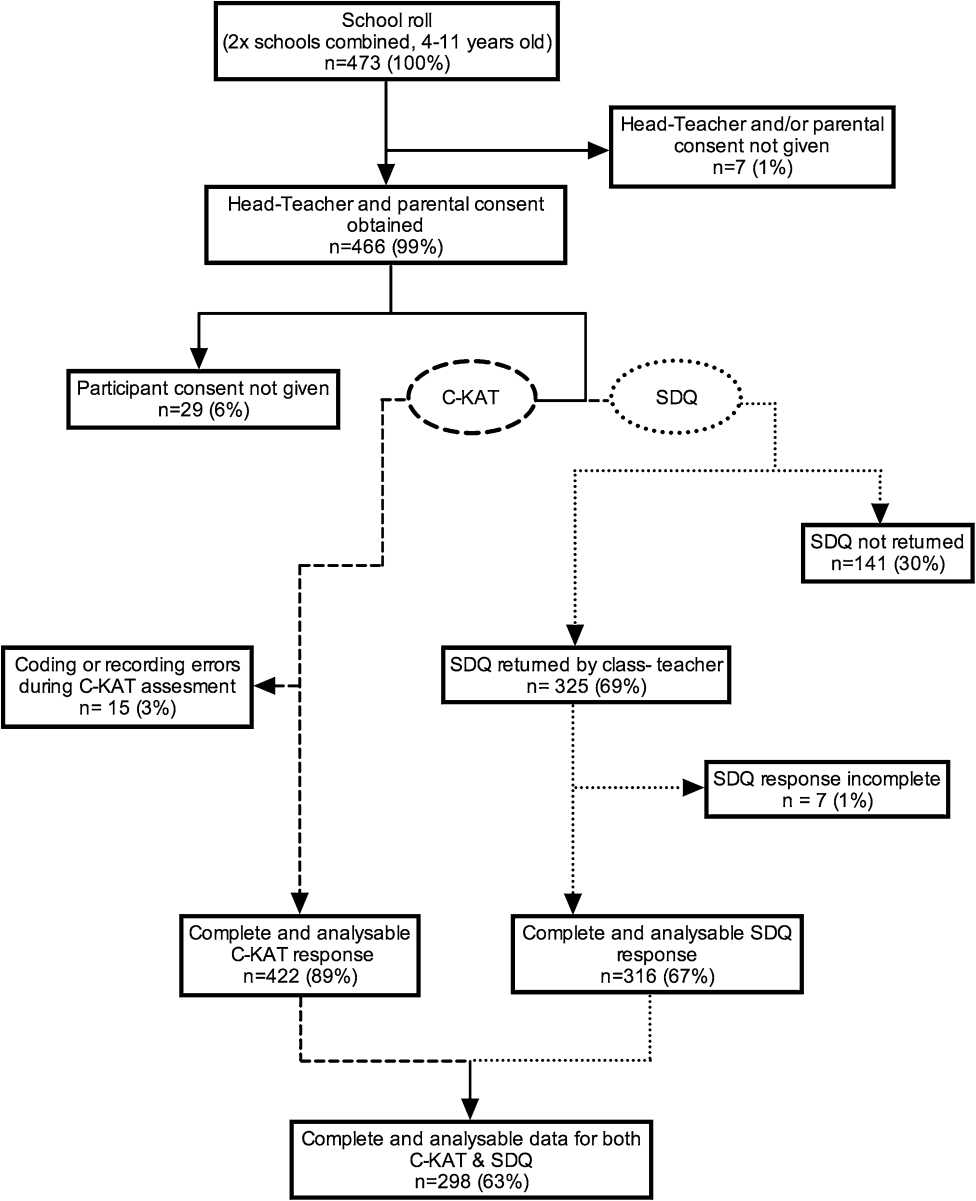
Flow diagram illustrating losses to attrition and exclusion from school roll to final sample of 298 participants. Notes: *C-KAT* Clinical Kinematic Assessment Tool; *SDQ* Strengths and Difficulties Questionnaire

All students aged 4–11 years old (*n* = 473) in two mainstream primary schools in West Yorkshire (UK) were invited to participate. Head-teacher and parent/guardian permission was granted in 466 of these cases (99 %; mean age = 7 years 6 months, range 4 years 0 months–11 years 11 months; 216 male, 250 female). An assessment of mental health and manual coordination was subsequently obtained for 298 of these students (63 %; mean age = 7 years 8 months, range 4 years 0 months–11 years 11 months; 136 males, 162 females). Figure 1 presents a flow diagram illustrating attrition and exclusion, whilst Table 1 gives a description of the final sample’s characteristics by age group. The study received approval from the University of Leeds Ethics Committee and was carried out in accordance with the ethical standards laid down in the 1964 Declaration of Helsinki and its later amendments.

**Table 1:** Descriptive statistics for gender and CKAT and SDQ response stratified by age for (A) the whole sample and (B) excluding participants with ‘Abnormal’ (high) SDQ Total Difficulty Scores (TDS)

	Total sample	4–5 years	6–7 years	8–9 years	10–11 years
(A) Whole sample					
*n*	298	76	91	72	59
Gender^a^					
Male	136 (46 %)	27 (36 %)	46 (51 %)	36 (50 %)	27 (46 %)
Female	162 (54 %)	49 (64 %)	45 (49 %)	36 (50 %)	32 (54 %)
CKAT response					
Overall battery score					
Mean (S.D)^b^	−0.02 (0.82)	1.00 (0.89)	−0.11 (0.38)	−0.44 (0.28)	−0.68 (0.20)
SDQ response					
Total difficulty					
Median (Range)	8 (0–27)	11 (1–27)	6 (0–27)	6 (0–21)	9 (0–24)
%Abnormal^c^	16 %	30 %	10 %	11 %	15 %
Internalising					
Median (Range)	5 (0–19)	6 (0–18)	4 (0–17)	4.5 (0–15)	6 (0–19)
Externalising					
Median (Range)	3 (0–16)	5 (0–16)	2 (0–13)	1 (0–12)	3 (0–12)
(B) Sample excluding Abnormal TDS responses					
*n*	249	53	82	64	50
Gender^a^					
Male	111 (45 %)	18 (34 %)	42 (51 %)	30 (47 %)	21 (42 %)
Female	138 (55 %)	35 (66 %)	40 (49 %)	34 (53 %)	29 (58 %)
CKAT response					
Overall battery score					
Mean (Std. Dev)^b^	−0.15 (0.65)	0.70 (0.69)	−0.10 (0.39)	−0.46 (0.27)	−0.72 (0.17)
SDQ response					
Total difficulty					
Median (Range)	6 (0–15)	9 (1–15)	5 (0–15)	5 (0–15)	7 (0–15)
Internalising					
Median (Range)	3 (0–14)	3 (0–12)	3 (0–14)	3.5 (0–14)	3.5 (0–13)
Externalising					
Median (Range)	2 (0–13)	3 (0–13)	2 (0–11)	1 (0–12)	2 (0–9)

^a^Denominators for percentages are each column’s *n* (see first row within each sub-table)

^b^Overall Battery score represents an average of three *z*-score transformed subtest performances. *z*-score transformation calculated relative to all valid C-KAT responses recorded (see Fig. 1, *n* = 422)

^c^UK norms for 5 to 15-year-old [61] estimate that Total Difficulty Scores will classify approximately 10 % of a community sample as scoring in the ‘Abnormal’ range (>15)

### Materials

#### Mental health materials

A brief, teacher-completed, behavioural screening questionnaire was used to assess children’s mental health: the Strengths and Difficulties Questionnaire (SDQ) [50]. This standardised questionnaire comprises five subscales (emotional symptoms; conduct problems, peer problems, hyperactivity and prosocial behaviour), each containing five items. These subscales are intended to assess core symptoms associated with common childhood psychiatric disorders [51]. Individual items on the SDQ ask about the frequency with which children exhibit both positive and negative attributes and because of this it is considered a particularly appropriate instrument for assessing mental health in community settings (i.e., in research where the majority of the sample is unlikely to demonstrate psychosocial problems [51]).

A Total Difficulties Score (TDS) for the SDQ is calculated by summing the scores for the responses to four of these five subscales, excluding the prosocial. A UK-based epidemiological study (*n* = 18, 415, 5–16 years old) has demonstrated that this TDS outcome is a meaningful dimensional measure of child psychopathology; the odds of clinical diagnosis of mental health problems increasing linearly with each one-point increase in the TDS [39]. A systematic review of evidence from epidemiological studies employing the SDQ has further supported its validity and reliability as a screening tool for assessing the prevalence of childhood mental health difficulties in community-based samples [52].

There is disagreement over the precise factor structure of the SDQ, with evidence from empirical factor analysis often contradicting the grouping of items into the five originally proposed subscales [53–56]. However, in low-risk community-based samples, evidence suggests that it is most meaningful (following analysis of the TDS outcome) to look at second-order Internalising (a sum of emotional and peer subscale scores) and Externalising (a sum of hyperactivity and conduct subscale scores) as nested outcomes [55, 57].

#### Manual coordination materials

To assess fine-motor control, an objective, computerised assessment was used: the Clinical-Kinematic Assessment Tool (C-KAT) [47, 58–60]. The C-KAT battery comprises three subtests (Tracking, Aiming and Tracing). On each subtest visual stimuli are presented on a tablet computer screen (Toshiba Portege M700-13P tablet, screen 260 × 163 mm, 1200 × 800 pixels, 60 Hz refresh rate) that participants must interact with using a hand-held stylus. The digitiser measures the planar position of the stylus at a rate of 120 Hz, allowing precise measurements of complex hand movements to be reliably captured. Previous research [47] demonstrated that these measurements have comparable temporal and spatial accuracy to those recorded using a ‘gold standard’ laboratory-based motion-capture (NDI Optotrak) system. Flatters et al. [58] provide a detailed description of the subtests and the specific outcome measures that each records. In brief, the Tracking subtest requires participants to keep the stylus tip as close as possible to the centre of a dot as it moves around the screen in a ‘Figure of 8’ pattern at increasing speed. The primary outcome for this subtest is the average distance between the stylus tip and the centre of the dot across the duration of the test (termed ‘Tracking Error’). The Aiming subtest has participants producing a series of aiming movements with the stylus, moving between sequentially presented targets appearing at equidistant pseudo-random locations on the screen. This subtests’ primary outcome is the average time it takes to arrive at each target measured from the point they arrived at the previous one (termed ‘Movement Time’). The Tracing subtest has participants tracing along a series of abstract shaped paths between designated start and finish points. Participants are asked to try and stay within guidelines (5 mm apart) that demarcate the paths. An accuracy measure is calculated for each trace, which is the average minimum distance between stylus tip and the centre-line of a path for the duration of that trace. This value is adjusted for the time taken to produce the trace and the median of these adjusted values for all traces is the primary outcome measure (termed ‘adjusted path accuracy).

### Procedure

Within a 2-month period at the end of the school year (May–July, 2012), classroom teachers were asked to complete an SDQ on each participating student within their class. During the same period the research team visited participating schools to conduct the manual coordination assessments. For this assessment participants were seated at a table with the tablet placed in front of them, 10 cm in from the table edge, with the screen presented horizontally (in landscape orientation). This made the interactions with the tablet similar to writing with a pen on paper. In total, the C-KAT battery took approximately 10–15 min to complete, enabling a brief but objective and precise assessment of manual ability in a large community-based sample. Performance on the primary outcome measure for each of the three subtests of the C-KAT was reciprocally transformed to normalise distribution within the sample, then converted into standardised units (*z*-scored). These *z*-score values had their sign inverted, to revert these scoring scales to the direction they ran in pre-reciprocal transform, before they were averaged across to give an overall battery score [C-KAT battery score].

## Results

All analyses were conducted in R (version 3.10.0, R Development Core Team, 2014).

### Preliminary data exploration

#### Mental health measures

Descriptive statistics (Table 1) indicated a higher than typical proportion of children within this community sample had a Total Difficulty Scores (TDS) that exceeded the threshold for being classified as ‘Abnormal’ (>15 out of 40). UK national norms [61] suggest approximately 10 % of a community-based sample should score above this threshold and that doing so is associated with a 15-fold increase in the odds of the child being independently diagnosed with a psychiatric disorder [62]. Stratifying for age, the frequency of abnormally high scores in this particular sample was found to be markedly elevated above this percentage in both the eldest and youngest aged participants (see Table 1). Descriptive statistics also indicated that the TDS, Internalising and Externalising subscale scores were all positively skewed in their distributions. Cronbach’s α indicated acceptable internal reliability for each of these scales (*α*_TDS_ = 0.85; *α*_Internalising_ = 0.79; *α*_Externalising_ = 0.88).

#### Manual coordination measures

C-KAT battery score was contrasted (using an independent *t* tests) between participants with a complete SDQ response also available and those without. No significant group differences were found, *t*(315.527) = 1.54, *p* = 0.124; with the effect-size for the mean-difference also small (*r* = 0.086), This suggested manual coordination did not differ systematically between cases included in subsequent analysis (*n* = 298) versus those excluded for lacking an SDQ response (*n* = 124).

### Confounding factors

Kendall’s tau correlation coefficients (Table 2) computed using robust bootstrap resampling techniques (2000 re-samples specified) revealed weak but statistically significant (*p* < 0.05) associations between gender and each of the SDQ subscales (i.e., Internalising and Externalising). The TDS outcome was not found to significantly correlate with gender because the relationships between gender and each of the two subscales (which combine to make the omnibus TDS score) ran in opposing directions. Correlational analysis also revealed Age was statistically significantly associated with the TDS and Externalising subscale score, respectively (see Table 2). No significant relationship (*p* = 0.059) was observed between age and the Internalising subscale score. These patterns of association remained similar even after excluding individuals whose SDQ Total Difficulty Scores exceeded the threshold for categorisation as ‘Abnormal’ (see Table 2).

**Table 2:** Kendall’s tau correlation coefficients for age and gender against SDQ outcomes, with and without participants with ‘Abnormal’ (high) SDQ Total Difficulty Scores (TDS) excluded

	*τ*	95 % CI^a^	*p* value
Total Difficulty Score			
Gender			
Whole sample (*n* = 298)	−0.073	−0.166 to 0.020	0.135
Excluding Abnormal TDS (*n* = 249)	−0.072	−0.177 to 0.032	0.181
Age			
Whole sample (*n* = 298)	−0.145	−0.222 to −0.066	<0.001
Excluding Abnormal TDS (*n* = 249)	−0.103	−0.189 to −0.017	0.019
Internalising subscale			
Gender			
Whole sample (*n* = 298)	−0.176	−0.269 to −0.082	<0.001
Excluding Abnormal TDS (*n* = 249)	−0.200	−0.299 to −0.100	<0.001
Age			
Whole sample (*n* = 298)	−0.076	−0.166 to 0.011	0.059
Excluding Abnormal TDS (*n* = 249)	−0.028	−0.124 to 0.067	0.528
Externalising subscale			
Gender			
Whole sample (*n* = 298)	0.105	0.010 to 0.201	0.036
Excluding Abnormal TDS (*n* = 249)	0.148	0.042 to 0.254	0.008
Age			
Whole sample (*n* = 298)	−0.165	−0.239 to −0.091	<0.001
Excluding Abnormal TDS (*n* = 249)	−0.119	−0.204 to −0.031	0.009

^a^ *CI* confidence intervals, calculated using bootstrap resampling (2000 resamples)

### Regression analyses

Having identified gender and age as additional predictors of SDQ response, a two-step hierarchical approach was followed in all subsequent (logistic and linear) regression analyses. That is, gender and age effects were accounted for prior to estimating the unique contribution manual coordination skills made to predicting SDQ responses. For a given SDQ outcome (e.g., TDS), a baseline (‘Step 1’) regression model was generated that specified the SDQ outcome as the dependent variable and gender and age as predictor variables. A second (‘Step 2’) regression model was then generated that was identical to the Step 1 model, except that it also included manual coordination skills (C-KAT battery score) as an additional predictor. Goodness of fit was then compared between the Step 1 and 2 regression models to determine whether inclusion of the additional motoric predictor significantly increased the explanatory power of the model.

#### Logistic regression analysis of SDQ Total Difficulty Score

The Total Difficulty Score (TDS) for the SDQ was specified as an outcome variable and categorised dichotomously, with an ‘Abnormally’ high (i.e., >15 out of 40) score on this scale being used as the criteria for defining mental health difficulties [62]. Hierarchical logistic regression analysis of this binary outcome indicated that an initial model, including only age and gender as predictors, was better than chance at predicting mental health difficulties (χ^2^ (2) = 9.53, *p* = 0.009). However, inclusion of C-KAT battery score as an additional predictor at Step 2 further improved model fit (χ^2^ (1) = 25.99, *p* < 0.001).

The odds ratios for the individual predictors included in the Step 2 model indicated that gender was not a significant predictor [OR (95 % Confidence Interval) = 1.50 (0.77–2.93)], whilst increased age was associated with a marginally increased risk [OR (95 % Cl) = 1.43 (1.09–1.88)] of mental health difficulties (as indexed by the SDQ). The direction of this finding contradicts the results of the earlier correlational analysis of age’s relations with SDQ Total Difficulty Score (Table 2). For every one-unit increase in C-KAT battery score (reduction in performance by 1SD), the odds of having a diagnosable psychiatric disorder increased almost sixfold [OR (95 % Cl) = 5.93 (2.90–12.87)].

#### Linear regression analysis of SDQ Total Difficulty Score

A square-root transformation was applied to the SDQ Total Difficulty Score (√TDS) prior to it being analysed as a continuous outcome variable. This ensured normally distributed residuals and homogeneity of variance in the subsequent models. Inclusion of C-KAT battery score as an additional predictor variable in step 2 of this hierarchical modelling significantly improved the amount of variance in √TDS explained (see Table 3, sections A and B). This was true irrespective of whether children with categorically ‘abnormal’ TDS scores (>15) were excluded from the analysed sample [whole sample: *F*(1, 294) = 55.26, *p* < 0.001; restricted sample: *F*(1, 245) = 25.65, *p* < 0.001].

**Table 3:** Multiple linear regression model for SDQ square-root Total Difficulties Score (√TDS) score predicted by gender, age and C-KAT battery (manual coordination) performance for (A) the whole sample and (B) excluding participants with ‘Abnormal’ (high) SDQ Total Difficulty Scores (TDS)

Predictor	Δ*R* ^*2*^	*b* [95 % CI]^a^	*β*	*p* value
(A) Whole sample (*n* = 298)				
Step 1	0.05			
Constant		3.74 [3.23, 4.23]		<0.001
Age		−0.12 [−0.05, −0.18]	−0.22	<0.001
Female		−0.24 [−0.50, 0.02]	−0.10	0.074
Step 2	0.15			
Constant		1.47 [0.77, 2.25]		<0.001
Age		0.18 [0.08, 0.27]	0.34	<0.001
Female		−0.25 [−0.50, −0.01]	−0.11	0.040
C-KAT battery		0.89 [1.12, 0.65]	0.68	<0.001
(B) Excluding Abnormal TDS (*n* = 249)				
Step 1	0.03			
Constant		3.07 [2.61, 3.50]		<0.001
Age		−0.07 [−0.12, −0.02]	−0.15	0.015
Female		−0.20 [−0.45, 0.03]	−0.10	0.105
Step 2	0.09			
Constant		1.58 [0.83, 2.26]		<0.001
Age		0.13 [0.04, 0.22]	0.27	0.009
Female		−0.20 [−0.43, 0.05]	−0.10	0.095
C-KAT battery		0.61 [0.39, 0.85]	0.52	<0.001

^a^ *CI* confidence intervals, calculated using bootstrap resampling (2000 resamples)

The estimated total amount of variance explained by the Step 2 model was 20 % [*R*^2^ (95 % CI) = 0.20 (0.13–0.28)], with this falling to 12 % if analysing the ‘Restricted’ sample that excluded children with a categorically Abnormal TDS score [R^2^ (95 % CI) = 0.12 (0.06–0.19)]. The amount of unique variance in √TDS explained by including manual coordination as a predictor (Δ*R*^*2*^ from the Step 1 to the Step 2 model) was always statistically significant but somewhat reduced in size by excluding children with a categorically Abnormal TDS score. Δ*R*^*2*^ equalled 0.15 in the ‘Whole’ sample but was 0.09 in the ‘Restricted’ sample (see Table 3, sections A and B).

#### Regression analysis of SDQ subscale scores

To explore relationships between the SDQ and manual coordination further separate linear regression analyses that specified the Internalising and Externalising subscale scores for the SDQ, respectively, as continuous dependent variables were conducted. As with the TDS, it was necessary to square root transform these variables prior to regression analysis. However, whilst this did improve the distribution of residuals in the subsequent regression models (compared to using uncorrected values) a degree of skew and heteroscedasticity was still evident. Consequently a more robust regression technique, which relaxed these assumptions, was employed and a Wald test was used to test change in goodness of fit between ‘Step 1’ and ‘Step 2’ hierarchical models (using *lmrob* and *anova.lmrob* from R package *Robustbase* [63]).

When (square-root transformed) *Internalising subscale* score was specified as the dependent variable regression analysis produced a similar pattern of results to those already presented in the “Regression analysis of SDQ Total Difficulty scores” section. Including C-KAT battery score as an additional predictor significantly increased the amount of variance the regression model explained in this outcome (whole sample Robust Wald test (1, 295) = 44.77, *p* < 0.001; restricted sample Robust Wald test (1, 246) = 25.96, *p* < 0.001). The amount of variance in this outcome that the C-KAT battery score explained (see Table 4) was similar to the amount of TDS variance it had previously been estimated to explain. This comparison was true both before and after excluding children with Abnormal TDS scores from the analysed sample (whole sample Δ*R*^*2*^ = 0.13; restricted sample Δ*R*^*2*^ = 0.08).

**Table 4:** Robust multiple linear regression model for SDQ Square-root Internalising subscale (√Internalising) score predicted by gender, age and C-KAT battery (manual coordination) performance for (A) the whole sample and (B) excluding participants with ‘Abnormal’ (high) SDQ Total Difficulty Scores (TDS)

Predictor	Δ*R* ^*2*^	*b* [95 % CI]^a^	*β*	*p* value
(A) Whole sample (*n* = 298)				
Step 1	0.07			
Constant		2.97 [2.27, 3.56]		<0.001
Age		−0.09 [−0.16, <−0.01]	−0.16	0.001
Female		−0.58 [−0.87, −0.28]	−0.24	<0.001
Step 2	0.13			
Constant		0.75 [−0.12, 1.75]		0.074
Age		0.20 [0.08, 0.31]	0.38	<0.001
Female		−0.59 [−0.88, −0.34]	−0.25	<0.001
C-KAT battery		0.86 [0.61, 1.10]	0.65	<0.001
(B) Excluding Abnormal TDS (*n* = 249)				
Step 1	0.07			
Constant		2.41 [1.74, 3.07]		<0.001
Age		−0.05 [−0.12, 0.03]	−0.09	0.245
Female		−0.58 [−0.86, −0.29]	−0.27	<0.001
Step 2	0.08			
Constant		0.82 [−0.17 1.80]		0.074
Age		0.16 [0.04, 0.28]	0.32	0.004
Female		−0.57 [−0.84, −0.28]	−0.26	<0.001
C-KAT battery		0.64 [0.39, 0.92]	0.50	<0.001

^a^ *CI* confidence intervals, calculated using bootstrap resampling (2000 resamples)

Similarly, in relation to the (square-root transformed) *Externalising Subscale* Score, analysis found that including C-KAT battery score as a predictor significantly increased the amount of variance explained by the regression model (whole sample Robust Wald Test (1, 295) = 28.55, *p* < 0.001; restricted sample Robust Wald Test (1, 246) = 11.01, *p* < 0.001). However, the amount of variation in the Externalising Subscale score that C-KAT was estimated to explain was somewhat smaller than for the previous analyses (see Table 5; whole sample Δ*R*^*2*^ = 0.08; restricted sample Δ*R*^*2*^ = 0.05).

**Table 5:** Robust multiple linear regression model for SDQ Square-root Externalising Subscale (√Externalising) score predicted by gender, age and C-KAT battery (manual coordination) performance for (A) the whole sample and (B) excluding participants with ‘Abnormal’ (high) SDQ Total Difficulty Scores (TDS)

Predictor	Δ*R* ^*2*^	*b* [95 % CI]^a^	*β*	*p* value
(A) Whole sample (*n* = 298)				
Step 1	0.06			
Constant		2.19 [1.70, 2.58]		<0.001
Age		−0.10 [−0.15, −0.04]	−0.22	<0.001
Female		0.25 [−0.01, 0.49]	0.12	0.051
Step 2	0.08			
Constant		0.59 [−0.17, 1.46]		0.112
Age		0.11 [<−0.01, 0.20]	0.23	0.022
Female		0.25 [0.03, 0.50]	0.12	0.039
C-KAT battery		−0.62 [−0.85, −0.31]	−0.55	<0.001
(B) Excluding Abnormal TDS (*n* = 249)				
Step 1	0.05			
Constant		1.65 [1.74, 2.61]		<0.001
Age		−0.06 [−0.15, −0.05]	−0.14	0.027
Female		0.33 [0.00, 0.49]	0.17	0.009
Step 2	0.05			
Constant		0.59 [−0.22, 1.42]		0.139
Age		0.08 [0.01, 0.20]	0.18	0.119
Female		0.33 [0.01, 0.48]	0.18	0.006
C-KAT battery		0.43 [0.34, 0.88]	0.40	0.001

^a^ *CI* confidence intervals, calculated using bootstrap resampling (2000 resamples)

## Discussion

In a study investigating the relationships between symptoms of mental health problems and motor control in a typical population of primary school children we found that manual coordination skills predicted approximately 15 % of the concurrent variation in symptoms of childhood mental health disorders, after adjusting for age and gender effects. A positive relationship was indicated, with poorer (increasingly inaccurate) manual coordination associated with more frequent mental health problems. A categorical relationship between coordination and the likelihood of mental health problems was also observed, replicating the findings of earlier population-based research [31–35]. These findings are noteworthy for three reasons.

First, they complement previous evidence of motor impairments co-occurring alongside other mental health problems in clinically referred samples [14–16] by demonstrating a similar relationship in the non-clinical population. Thus, these results confirm that co-occurring difficulties are unlikely to be solely attributable to ascertainment and referral bias.

Second, we found that an association persisted even after we removed those with high TDS scores from the population, indicating that there exists a broader dimensional relationship between motor function and other aspects of psychological development, with co-occurrence only representing the tail of a continuum. The finding corroborates previous suggestions that motor skill acquisition is a critically important factor within childhood development [10, 11] and underscores previously expressed concerns that this is often neglected within education and health service provision [27, 64].

Third, further analysis of the SDQ subscales suggested manual coordination is associated with both internalising and externalising behaviours. This relationship between manual coordination and both SDQ subscales is not altogether surprising if we consider the wide range of mental health problems which commonly co-occur alongside DCD [10, 11]. Such a finding also supports the notion that all aspects of mental health in young people are reliant on intact and healthy neurodevelopment. The association between neurological disorder and mental health problems has long been recognised (e.g., since the Isle of Wight studies in the 1960s and 1970s [65]) but here we demonstrate a dimensional association, indicating that it extends more broadly across population and function.

A relationship on the externalising sub-scale is consistent with the high-levels of co-occurrence noted between DCD and ADHD symptomology [7, 34, 36]. Epidemiological research indicates the SDQ’s hyperactivity subscale (one of the two scales contributing to the externalising score) has reasonable sensitivity and specificity as a population-based screening tool for ADHD [66, 67]. Conversely, relationships with the Internalising subscale (comprising emotional and peer problems) may reflect the well-documented associations between motor coordination problems and internalising disorders such as anxiety and depression [41, 68, 69]. We found some evidence for the strength of manual coordination’s relationship with internalising behaviours (Δ*R*^*2*^) possibly being greater than externalizing behaviours. However, caution must be exercised in making this inference, given generalisability issues with these subscales’ regression models. Further investigation is required to see if this effect can be replicated. Such a difference may be symbolic though of motor control’s relationship with internalising and externalising behaviours, respectively, being qualitatively distinct. Further support for this view comes from Emck et al’s [8] review of motor difficulties in children with behavioural, emotional and pervasive developmental disorders, in which they observe that children with behavioural difficulties (i.e., more externalising symptomatology) tend to overestimate their motor-competence, whilst children with emotional (i.e., more internalising symptomatology) and pervasive disorders tend to have self-perceived motor-incompetence.

### Implications

Measures of motoric function may simply be indices of neurodevelopmental integrity, which itself is a predictive factor for mental health across the whole population. However, there is also a question as to what extent motor abilities contribute to the development of mental health. Our correlational study cannot speak to causality but a number of plausible mechanisms have been postulated, which would explain motor functions’ associations with other aspects of neurodevelopment. An underlying genetic basis for motor difficulties’ co-occurrence with attentional and anxiety problems (without causality in either direction) has been reported by Moruzzi et al. [37]. Thus it may be that co-occurrence is explained by certain genetic [43] or environmental [70] factors that pre-dispose children to a range of developmental disorders. Additionally (or alternatively) more specific aetiological pathways may also underpin specific types of co-occurrence. For example, difficulties within language and motor-control are thought to possibly reflect an underlying deficit in children’s ability to achieve automaticity in the course of their procedural learning, which then causes deficits in both these areas of function [71, 72]. On the other hand, recent experimental research indicates that fine-motor deficits are not associated with reading disability if oral language impairments are taken into account, implying a generalised deficit relating to automatising procedural learning cannot wholly explain co-occurrence [22]. Also worth considering is that a number of aspects of psychological development may utilise motor abilities and benefit from motor rehearsal in development. Recent theories of embodied cognition consider the neural bases of motoric function to be critical to functions which depend upon motor skills for their expression, whether this is executive function, the expression of emotion or its role in memory [73] or imitation. Finally, it has been suggested that motor difficulties specifically may precipitate anxious and depressive symptomatology [38, 41], possibly due to the limitations that motor impairment places on development in other areas of function (e.g., limiting social interaction, academic progress).

Irrespective of causal mechanisms, our results suggest that a more holistic approach to evaluating psychological developmental at a population level is required, including assessments of motor skill alongside other facets of development. For example, current brief screening tools for assessing child mental health, such as the SDQ [50], often do not include an assessment of motor skill. Meanwhile, large-scale epidemiological studies explicitly examining co-occurrence in developmental disorders have often focused their efforts on understanding social, emotional and cognitive aspects of child development at the expense of assessing motor skills with equal rigor [43]. These points run contrary to motor function being acknowledged alongside other Disorders of Psychological Function in the ICD-10 disease classification framework [3]. Indeed, a more comprehensive and inclusive perspective is required in future research if it is to adequately inform implementation of a more flexible ‘patient centred’ approach to diagnosing and supporting individuals with developmental disorders [23, 74, 75].

### Limitations

The study design estimated participants’ motor-function via a tablet-based unimanual coordination task [47, 58]. This assessment enabled a highly objective measurement to be obtained from a large community-based sample of children but only in relation to a specific subset of motor skills (i.e., coordinative ability for manipulating a handheld stylus with one hand). Previous research, using more subjective assessment tools, has suggested the degree of the co-occurrence between DCD and ADHD varies depending on diagnostic sub-type, with the strongest association being observed between Inattentive sub-type ADHD and fine-motor skill deficits specifically [36]. Therefore, further investigation is necessary to confirm whether the current findings alter when a wider set of objectively assessed motor functions is assessed (e.g., gross and fine motor skills). More broadly, it would also be interesting to assess a wider set of psychological functions (e.g., motor skill, attention, working memory) using an equally objective but more comprehensive battery of computerised tests. This more detailed investigation would enable a study of the shared and individual contributions specific psychological processes make to specific mental health problems (e.g., DCD and ADHD). Theories of embodied cognition [73] would predict that such an investigation might not only reveal certain constructs (e.g., motor function) having a strong localised influence on battery sub-tests designed to measure this function specifically but also a more diffuse impact on tests considered to be relatively ‘pure’ indices of other cognitive constructs. For example, participants’ performance on a test designed to measure attention might be influenced to a lesser (but still significant) degree by the need to respond motorically. Vice versa, sustained attention and memory for instruction might affect a participant’s performance on a complicated subtest of motor coordination. Understanding this complex pattern of interactions is vital if we are to fully comprehend the mechanisms by which co-occurring mental health problems might arise.

It should also be noted that neither parent nor self-report SDQ responses were obtained from participants. Whilst the teacher-response version of the SDQ is considered the most reliable assessment tool out of the three [52], multi- compared to single-informant SDQ response is known to improve the instruments’ sensitivity for detecting cases of psychopathology [52, 70, 76]. In this study SDQ response was treated as a proxy measure of mental health problems and thus using a multi-informant approach may have improved its validity.

Future research might also look to control a wider range of potentially moderating factors such as IQ [31] and ethnicity [77]. This study did control for gender and age effects. Gender has been frequently observed in previous studies influencing SDQ response [56, 78, 79] but association between SDQ response and age warrant further investigation. On the one hand, the SDQ is indicated to be standardised for use in 4–16 year olds [50, 52]. For example, the UK norm data in relation to the teacher-report version (5–15 years old) confirms the validity of applying the cut-off for scoring responses as ‘Abnormal’ >15 in relation to its TDS metric [61], with this threshold having been shown to bear clinical significance across this age-range [62]. However, our data suggest mild associations between SDQ response and age in a UK sample, similar to more recent investigations showing TDS scores decreasing with age in Dutch [78], Finnish [80] and Chinese [56] children. These finding call into question the appropriateness of using the SDQ to categorically (rather than dimensionally) assess child mental health, particularly in children younger or older than 5 or 15 years of age (i.e., outwith the age of the original norms).

## Conclusions

This is the first study of its kind to utilise objective computerised methods to assess manual motor coordination with a high level of precision and relate this to psychopathology in a community sample. We have demonstrated the existence of a dimensional relationship between children’s manual coordination and other aspects of their psychological functioning and mental health. The finding of a significant result is remarkable given that only one aspect of motor skill was investigated (albeit with a sophisticated instrument capable of precise measurement) and related to a single informant response on a behavioural screening questionnaire. It hints at the potential strength of the underlying relationship and begs further longitudinal and cross-sectional research in nationally representative samples (exploring different aspects of motor control and utilising finer grained indices of child mental health). Our findings strongly suggest that greater consideration needs to be given to assessing and supporting motor as well as social, emotional and cognitive development in children, particularly in early childhood.
